# Inequalities in Mortality in the Asia-Pacific: A Cross-National Comparison of Socioeconomic Gradients

**DOI:** 10.1093/geronb/gbad193

**Published:** 2024-01-09

**Authors:** Kim Qinzi Xu, Jessica Yi Han Aw, Collin F Payne

**Affiliations:** School of Demography, Research School of Social Sciences, The Australian National University, Canberra, Australian Capital Territory, Australia; School of Demography, Research School of Social Sciences, The Australian National University, Canberra, Australian Capital Territory, Australia; School of Medicine and Psychology, College of Health and Medicine, The Australian National University, Canberra, Australian Capital Territory, Australia; School of Demography, Research School of Social Sciences, The Australian National University, Canberra, Australian Capital Territory, Australia; Harvard Center for Population and Development Studies, Harvard T.H. Chan School of Public Health, Cambridge, Massachusetts, USA; (Social Sciences Section)

**Keywords:** Asia-Pacific, Cross-national comparison, Mortality, Social inequality, Socioeconomic status

## Abstract

**Objectives:**

Social inequalities in mortality are poorly studied in much of the Asia-Pacific. Using data from harmonized nationally representative longitudinal health and aging surveys our study systematically assesses mortality disparities across 3 standardized measures of socioeconomic status in 7 Asia-Pacific countries.

**Methods:**

We used data from multiple waves of 7 representative sample surveys: the Health, Income and Labour Dynamics in Australia survey, China Health and Retirement Longitudinal Study, the Indonesian Family Life Survey, the New Zealand Health, Work and Retirement survey, the Korean Longitudinal Study on Ageing and the Health, Aging and Retirement in Thailand survey, and the Japanese Study of Aging and Retirement. We use Cox proportional hazards modeling to examine how the hazard of mortality differs across domains of social stratification including educational attainment, wealth, and occupational status across countries.

**Results:**

We found consistent and pervasive gradients in mortality risk in the high-income countries by all available measures of social stratification. In contrast, patterns of inequality in adult mortality in middle-income and recently transitioned high-income countries investigated varied depending on the measure of social stratification, with strong gradients by wealth but mixed gradients by education.

**Discussion:**

Analyzing social gradients in mortality in the Asia-Pacific shows that inequalities, especially wealth-based inequalities, in later-life health are present across the region, and that the magnitude of social gradients in mortality is overall larger in high-income countries as compared to middle-income countries.

In recent years, one of the major lines of inquiry in research on mortality patterns has centered on social inequalities in mortality and longevity. Wide gradients by socioeconomic status (SES) are seen across a variety of mortality, health, and well-being indicators. However, outside of high-income countries (HICs), the research evidence on social inequalities in mortality is piecemeal and inconsistent, and there is a lack of high-quality studies comparing social gradients in mortality (van Raalte, 2021).

This lack of comparable evidence on social inequalities in adult mortality is particularly problematic in the Asia-Pacific region. Across much of the region, growth in the population aged 50+ is now substantially outpacing total population growth. In tandem with these age-compositional shifts, life expectancies in the region have risen rapidly—increasing by a decade over the last 35 years and projected to rise by another 5 years by 2040 (United Nations, 2015). Supporting the health and well-being of these older populations is one of the largest challenges faced today by the countries in the Asia-Pacific, and one that will only grow in coming decades. However, the social and economic gradients in later-life mortality are poorly studied in much of the Asia-Pacific, and little existing research sought to understand how the relative magnitude of social gradients in mortality varies across Asia-Pacific populations.

Among HICs, a considerable body of literature focused on the demography and social epidemiology of health, has evolved to explore the role of socioeconomic status in individual well-being (Glymour et al., 2014; Link & Phelan, 1995). Substantial socioeconomic gradients have been observed in mortality and life expectancy: the wealthier and more educated individuals experience longer, healthier lives. Recent evidence suggests that these gradients have widened in recent years in a number of high-income contexts (Long et al., 2023; Olshansky et al., 2012). However, outside of Europe, North America, and other Anglo-Saxon countries, cross-national comparisons of social inequalities in mortality remain scarce.

Existing mortality studies in the Asia-Pacific are often confined to single-country analyses (Fujino et al., 2005; Ito et al., 2008; Jatrana & Blakely, 2014; Khang et al., 2004; Korda et al., 2020; Liang et al., 2000; Son et al., 2002; Y. Luo et al., 2015). These studies generally find that higher-SES individuals tend to experience lower mortality in China, South Korea, Australia, and New Zealand, although evidence of the social gradient in Japan is less clear. However, the SES measures used in these single-country analyses are not directly comparable, making it difficult to determine how the magnitude of social gradients in mortality compares across countries. Of the few cross-national studies from the Asia region, most include only high- or higher–middle-income countries such as Japan, Korea, and China (Sudharsanan et al., 2020; Wu et al., 2020). This scarcity of cross-national mortality studies in the Asia-Pacific is driven largely by a lack of harmonized population-level data linking SES to mortality outcomes. Further, existing studies have generally focused on single measures of SES such as educational attainment or occupation, rather than comparing multiple measures of SES (Fujino et al., 2005; Ito et al., 2008; Khang et al., 2004; Liang et al., 2000; Son et al., 2002; Sudharsanan et al., 2020; Y. Luo et al., 2015). Educational attainment has been the most common measure in previous work. However, using educational attainment as a single measure of SES may be inadequate in examining health in later life. Studies investigating education and later-life health outcomes have been unable to establish a causal pathway as this relationship is mediated through achieved SES which also includes other measures such as occupation and wealth (Chandola et al., 2006; Davey Smith et al., 1998). In addition, prior research has often found an inconsistent educational gradient in mortality between countries, suggesting that alternative measures of SES may be needed (Sudharsanan et al., 2020).

In the limited studies that include monetary measures of SES, most have focused on income as a measure of economic resources (W. Luo & Xie, 2014). However, when studying older populations, retirement often leads to reduced income, meaning that a measure of cumulative economic resources may be a better indicator of socioeconomic position (Kaplan et al., 1987). Among older populations, wealth may be a better measure of economic resources compared to income, as it reflects accumulation from previous generations and throughout an individual’s life course and is more stable over time (Christensen et al., 2014; Duncan et al., 2002; Nowatzki, 2012; Rodrigues et al., 2018) Combined wealth at a household level can reflect a family’s ability to maintain a particular living standard and their overall financial security (Spilerman, 2000). Although current evidence suggests wealth can explain a range of health outcomes in a population (Pollack et al., 2007), little existing work has compared wealth-based disparities in mortality in a cross-national context, and no studies of wealth-based inequality in mortality exist in the Asia-Pacific.

There are reasons to suspect SES differences in late-life mortality across middle-income countries (MICs) and recently transitioned HICs in the Asia-Pacific may not follow the inverse SES patterning observed in established HICs. A substantial portion of these gradients is thought to be explained by differences in health behaviors—that is, rates of smoking, obesity, and low physical activity (Nandi et al., 2014; Stringhini et al., 2011). In established HICs, these unhealthy behaviors tend to follow an inverted relationship with SES, meaning that they are more common among the poor than the rich (Pongiglione et al., 2015). In contrast, these behaviors follow an inverted or less-consistent SES patterning in many MICs and recently transitioned HICs in the Asia-Pacific (Ahmed et al., 2009; Sudharsanan, 2017). This may lead to higher-SES groups having higher mortality from lifestyle-associated diseases such as heart disease, diabetes, and hypertension.

Although the social patterning of health behaviors may not be as clear cut in MICs and recently transitioned HICs, these behaviors are not the only way that SES can influence later-life mortality. A number of potential pathways linking SES to health and mortality outcomes have been investigated in prior research, although much of the evidence base has centered on HICs (van Raalte, 2021). SES has been theorized as a “fundamental cause” of disease and mortality that reproduces health inequalities across contexts, as the resources available to an individual can act to shape health-related exposures in protective ways; likewise, the lack of resources can have a deleterious impact on health-related exposures (Link & Phelan, 1995; Phelan & Link, 2005). Individuals at lower levels of SES are also subject to greater exposure to both acute and chronic stress, both of which are closely linked to chronic inflammation, cardiovascular, and cardiometabolic disease (Pearlin et al., 2005). These socially derived sources of cumulative inequality and stress may play a substantial role in determining patterns of mortality inequality.

To the best of our knowledge, no existing research has compared social inequalities in mortality in the Asia-Pacific region in depth. In this analysis, we examine how the hazard of mortality differs across domains of social stratification including educational attainment, wealth, and occupational status in a number of Asia-Pacific countries. We seek to understand the patterning of social inequalities in mortality in the region, exploring inequalities by wealth, schooling attainment, and occupational status. There is considerable heterogeneity across and within countries in the Asia-Pacific region. Our analyses include four established and recently transitioned HICs including two Anglo-Saxon (Australia and New Zealand) societies, two East Asian (Japan and South Korea) societies, and three MICs in East and Southeast Asia (China, Indonesia, and Thailand). Using data from harmonized nationally representative longitudinal health and aging surveys data, our study systematically assesses mortality disparities across these three standardized measures of SES in these seven Asia-Pacific countries. This research represents a substantial step forward in the scholarship on cross-national differences in the magnitude of social gradients in mortality.

## Data and Methods

### Data

We used data from multiple waves of seven representative sample surveys to explore SES gradients in mortality in the Asia-Pacific: the Health, Income, and Labour Dynamics in Australia (HILDA) survey, China Health and Retirement Longitudinal Study (CHARLS), the Indonesian Family Life Survey (IFLS), the New Zealand Health, Work and Retirement (NZHWR) survey, the Korean Longitudinal Study on Ageing (KLoSA) and the Health, Aging and Retirement in Thailand (HART) survey, and the Japanese Study of Aging and Retirement (JSTAR). The baseline sample of JSTAR included individuals from five pre-chosen municipalities, all of which are located in the eastern part of Japan. As such, caution is needed in the interpretation of our results pertaining to Japan, as JSTAR is not nationally representative (Ichimura et al., 2009). Information about each study’s sampling method and which waves were included are detailed in Supplementary Table 1. To focus on adult mortality, we restricted our sample to individuals 45 years above or the minimum sampling age if it was older than 45 years and excluded individuals who were missing age and or gender.

All of our analyses use survey weights, except our analyses of the HART (Thailand) survey where these weights were not available. Individuals without follow-up information were excluded from the analysis. For each data source, we used stabilized inverse probability weighting (IPW) to adjust for bias due to this loss to follow-up (Seaman & White, 2013). Weights were estimated using logistic regression. Our missingness model in IPW included a range of sociodemographic variables including age, gender, marital status, urban residence, educational attainment, household wealth, ever smoke, and ever had alcohol. The final analysis weight used is the survey weight multiplied by the IPW-based attrition weight (DuGoff et al., 2014; Payne & Wong, 2019).

To maximize the usable sample size in each survey, we used multiple imputation by chained equations to impute values for missing data. Missing data were most frequent in household wealth and occupation. We imputed missing values by predictive mean matching for wealth, and by multiple logistic regression for occupation. The missingness model—which included age, gender, marital status, urban residence, educational attainment, ever smoked, ever had alcohol, sampling strata, and cross-sectional weight—generated 20 data sets. We included the mean of these 20 imputed values in our analysis. The percentages of imputed missing values for each survey are presented in Table 2.

**Table 2.** Socioeconomic Distribution Across Countries

SES measure	Australia	China	Indonesia	Japan	New Zealand	South Korea	Thailand
Education							
No schooling	—	9,949 (41.4%)	6,002 (44.5%)	—	—	1,843 (16.5%)	130 (3.5%)
Primary (or less)	3,393 (40.5%)	6,345 (26.4%)	3,463 (25.7%)	1,798 26.4%)	2,613 (40.9%)	4,678 (41.9%)	3,114 (38.3%)
Secondary	3,267 (39.0%)	7,137 (29.7%)	3,042 (22.6%)	3,928 (57.7%)	3,180 (49.7%)	3,416 (30.6%)	494 (13.2%)
Tertiary	1,717 (20.5%)	601 (2.5%)	974 (7.2%)	1,089 (16.0%)	601 (9.4%)	1,234 (11.1%)	—
Education in years							
Low	2,793 (33.4%)	9,949 (41.4%)	6,002 (44.5%)	—	2,132 (33.4%)	4,696 (42.0%)	—
Middle	2,792 (33.3%)	6,345 (26.4%)	3,463 (25.7%)	—	2,131 (33.3%)	5,025 (45.0%)	—
High	2,792 (33.3%)	7,738 (32.2%)	4,016 (29.8%)	—	2,131 (33.3%)	1,450 (3.0%)	—
Occupation							
Manual	—	—	1,931 (15.3%)	—	—	4,124 (36.9%)	—
Farmers	2,437 (29.1%)^a^		4,073 (32.3%)			1,492 (13.4%)	
Lower nonmanual	2,181 (26.0%)^a^		5,538 (43.9%)			4,257 (38.1%)	
Upper nonmanual	3,759 (45.9%)^a^		1,067 (8.5%)			1,298 (11.6%)	

*Notes*: Occupation was not available in CHARLS (China), JSTAR (Japan), NZHWR (New Zealand), and HART (Thailand). CHARLS = China Health and Retirement Longitudinal Study; HART = Health, Aging and Retirement in Thailand; HILDA = Health, Income and Labour Dynamics in Australia; JSTAR = Japanese Study of Aging and Retirement; NZHWR = New Zealand Health, Work and Retirement; SES = socioeconomic status.

^a^Categories for HILDA occupation are low, middle, high.

### Measuring Mortality

Each survey included information on deaths occurring between sample waves reported by a family member during a follow-up wave of data collection. The HILDA survey was matched to the National Death Index in 2014 and the dates of death were added to the data files (Watson & Summerfield, 2014). A number of surveys including IFLS, KLOSA, and HILDA report the exact date of death for deceased individuals. HART, JSTAR, and NZHWR report year of death. CHARLS survey only provided information on whether the respondent was alive or deceased at the time of the interview, and hence we estimated the date of death as halfway between survey waves.

### Measuring Educational Attainment

Each survey included information on the respondents’ highest completed level of schooling. We classified individuals into four categories of schooling attainment: no formal schooling, primary schooling, secondary schooling (including lower and upper secondary schooling), and tertiary schooling according to the International Standard Classification of Education (ISCED; Statistics, 2012). Details of how these categorizations were specifically constructed for each country are included in Supplementary Table 2.

In some analyses, where there was insufficient sample size, not all group categories were included—that is, in the HILDA (Australia) data set, there were not enough older adults who received no formal schooling to estimate a coefficient, and similarly, in the HART (Thailand) data, there was insufficient sample to estimate a coefficient for those with tertiary schooling. We additionally note that these common groupings of schooling may not reflect similar levels of SES across contexts. For example, it is fairly common among older adults in Australia to be a university graduate, but in Indonesia, it is rare. Therefore, having a university degree in Indonesia may be a stronger indicator of high SES than in Australia. In our interpretation of results, we focus on the relative gradient in mortality across educational groups within each country, rather than direct comparisons between education groups across contexts. We further present results based on tertiles of years of schooling for the five countries where information on years of schooling was available.

### Measuring Household Wealth

We used measures of overall net household wealth through an asset index measure used by the U.S. Health and Retirement Survey (Hurd et al., 2016). This measure incorporated values of financial assets including savings, investments, pensions, rental income, nonfinancial assets including housing value, and other durable assets, minus all debts held at the household level. These measures have been generated by HRS sister studies including CHARLS, IFLS, JSTAR, and KLoSA. For HILDA and HART, we generated our own measures to ensure comparability. Detailed information on household wealth was not available in NZHWR. The items used to generate net household wealth are detailed in Supplementary Table 3.

We calculated the total value of net household assets in each country’s currency value. For each survey, we used the latest observed household wealth and divided these distributions into tertiles. Wealth tertiles were generated separately within 10-year age bands (45–54, 55–64, 65–74, 75–84, and 85+) to reduce the potential for age-related changes in wealth to affect our findings. For example, older individuals may draw down their wealth during retirement. Directly comparing the level of wealth of an individual in, say, their 80s, who has steadily drawn down their wealth in retirement, to an individual in, say, their 50s, who is actively building wealth, could be problematic. Our analytical approach focused on a relative (rather than absolute) measure of wealth to facilitate direct comparisons of social gradients in health across countries. This relative nature of this strategy does not assume direct comparability of the dollar value of wealth. Rather, the emphasis was on identifying how wide or narrow wealth gradients in mortality are within each country.

### Measuring Occupational Status

Three surveys (KLOSA, IFLS, and HILDA) reported information on respondents’ current primary occupation, or primary occupation during their working life for retirees and those unemployed at the time of the survey. For these surveys, we grouped occupations into four categories: upper nonmanual workers (including professionals and managers), lower nonmanual workers (e.g., clerical, service, sales workers, manual workers including craft and related trades workers), semiskilled and unskilled manual workers, and farmers. We categorized occupation groups according to the Erikson–Goldthorpe–Portocarero scheme, which was developed to facilitate international comparisons (Erikson et al., 2010). The classification of specific occupations is presented in Supplementary Table 4.

HILDA reported the Australian Socioeconomic Index 2006 (AUSEI06) of the respondents’ primary occupations. The AUSEI06 is a continuous scale ranging from 0 (low status) to 100 (high status), coded in accordance with the Australian and New Zealand Standard Classification of Occupations. In line with previous research, we derived three status groups by dividing AUSEI06 on the 25th percentile and median (D’Souza et al., 2005): the upper 50% included most managerial and professional occupations, whereas the lowest 25th percentile included most routine, manual, or elementary occupation. Individuals who had never worked were excluded from the analyses of occupational class.

## Method

We estimated SES differences in the hazard of mortality using a Cox proportional hazards regression, with indicator variables for education, wealth, and occupation groups. We used age as the time scale, given that age is a strong determinant of mortality risk (Thiébaut & Bénichou, 2004). We used the lowest levels of education, wealth, and occupation as reference groups. Consequently, the estimated coefficients indicated the hazard ratios (HRs; 95% confidence intervals [Cis]) for mortality relative to these SES groups. Because our aim was to observe SES patterning of mortality, all models were adjusted only for gender. We relaxed the assumption of proportionality by allowing the baseline hazard functions to differ by gender. To assess gender differences in gradients in mortality by the three measures of SES, we additionally repeated the analysis stratified by gender. All analyses include cross-sectional survey weights to weight to the national sample, except for analyses of the HART (Thailand) data where survey weights were unavailable. The age at death distribution is provided in Supplementary Table 5. All analyses were conducted in STATA 17 (College Station, TX).

## Results

Exploring the broad demographic changes occurring in the countries included in this study demonstrates the rapid pace of aging in the region. Over the 35 years between 2005 and 2040, the proportion of the population aged 50+ is projected to more than double in China, Indonesia, South Korea, and Thailand, and will increase by nearly 10% in Australia, New Zealand, and Japan (Supplementary Table 6). As of 2020 individuals aged 50+ make up more than a third of the populations of Australia, Japan, New Zealand, South Korea, and Thailand, and by 2040, this age group will account for more than 40% of the population in China, Japan, South Korea, and Thailand.

Table 1 presents the baseline sample size and characteristics of each survey included in this analysis. The median age of each survey varied from a low of 51 years in Indonesia to a high of 66 years in Thailand. All samples had more women than men, likely resulting from higher survivorship of women in older ages. Table 2 further presents the distribution of SES in each country.

**Table 1.** Baseline Sample Characteristics

Country	Survey sample size	Median age (IQR)	Percent female (%)	Number of deaths	Missing information (% of eligible)		
					Education (%)	Wealth (%)	Occupation (%)
Australia	10,151	55 (47–66)	53	686	0.1	2.8	1.6
China	24,032	56 (49–64)	51	2,068	0.1	17.9	n/a
Indonesia	13,481	51 (48–62)	53	1,749	0.5	2.2	11.4
Japan	6,813	63 (57–69)	52	152	0.5	0.1	n/a
New Zealand	6,394	60 (57–65)	54	703	2.2	n/a	n/a
South Korea	11,171	60 (52–70)	56	2,215	0.0%	0.4	7.7
Thailand	3,742	66 (57–76)	53	132	0.1%	3.4	n/a

*Notes*: IQR = interquartile range; n/a = not applicable.

To investigate how risk factors for mortality differed across measures of SES, we explored gradients in three health behaviors (ever a smoker, frequent drinking [drinking >4 days a week], and overweight/obese body mass index [BMI ≥ 25]). Supplementary Tables 7–9 present these relationships. To account for potential differences in age composition across different populations (which could affect comparability if health behaviors are associated with age), these comparisons are age standardized to the average age distribution of the populations under study (Preston et al., 2000). Although the level of smoking prevalence (Supplementary Table 7) differed across countries, we found that across all contexts, there was a clear social gradient in smoking behaviors. Wealthier and more educated individuals were less likely to have ever smoked, and in China, Indonesia, South Korea, and Thailand, women were substantially less likely to report ever smoking. We also note that the New Zealand survey only asked whether an individual was currently a smoker and not ever a smoker; the results of New Zealand smoking behaviors are thus not directly comparable to those from other countries.

Socioeconomic patterns of frequent drinking (Supplementary Table 8) were more mixed.

In Australia and New Zealand, frequent drinking followed an inverted gradient, where higher-SES individuals reported a higher frequency of using alcohol. In contrast, frequent drinking was less socially patterned in China and Japan, whereas in South Korea and Thailand, higher-SES individuals were slightly less likely to report frequent drinking. We note that frequent drinking was reported at much lower rates among women than men, a difference that was particularly pronounced in the Eastern and Southeastern Asian countries in the study. In addition, the IFLS (Indonesian) survey did not ask questions about alcohol use.

Similar to the gradients in frequent drinking, the social patterning of overweight or obese BMI (≥25) was somewhat mixed across the region (Supplementary Table 9). Overall, the level of overweight/obesity was substantially higher in Australia than in other countries (noting that BMI was not collected in the NZHWR-New Zealand study). In Australia and Japan, individuals with higher levels of schooling and higher wealth were less likely to be overweight or obese, although these gradients were not especially strong in Japan. In South Korea and Thailand, gradients in overweight/obesity were fairly minimal, although there was some evidence that tertiary-educated individuals in South Korea were less likely to be overweight or obese. In China and Indonesia, more advantaged individuals had substantially higher rates of overweight/obesity.

### Education

Table 2 provides the HRs (and 95% CIs) of mortality across different levels of educational attainment in the seven countries included in this analysis. Broadly, we found that more highly educated individuals have a clear mortality advantage. In all countries, a clear gradient emerged in later-life mortality risk based on schooling attainment—that is, even those not in the highest groupings of schooling attainment were still advantaged over those with the lowest schooling attainment. However, in two of the middle-income contexts in the study, only the highest group (secondary schooling in HART, tertiary in IFLS) seemed to convey significant advantages in terms of lower mortality. In Japan, secondary schooling was associated with significantly lower mortality compared to those with primary school education. However, those with tertiary schooling did not have a significant mortality advantage in relation to those with primary schooling.

We further stratified these analyses by gender, the results of which are presented in Tables 3 and 4. We found important gender differences in the relationship between education and mortality. First, we observed that tertiary schooling does not convey mortality advantages among Japanese men and South Korean women. Second, in Australia, New Zealand, and Indonesia, tertiary schooling was associated with markedly lower mortality among men than women as compared to those with the lowest levels of education. Third, in the three East Asian societies, that is, China, Japan, and South Korea, with the exception of tertiary educated in South Korea, we found steeper schooling gradients at all levels for women as compared to men, with evidence of a schooling advantage starting from primary schooling for women.

**Table 3.** Relationship Between Socioeconomic Measures and Mortality, Hazard Ratios (95% CI)

SES Measure	Australia	China	Indonesia	Japan	New Zealand	South Korea	Thailand
Education							
No schooling	—	Ref	Ref	—	—	Ref	Ref
Primary	Ref	**0.80 [0.70–0.93]**	1.11 [0.98–1.25]	Ref	Ref	0.91 [0.81–1.02]	0.97 [0.42–2.27]
Secondary	**0.72 [0.59–0.89]**	**0.65 [0.54–0.79]**	0.96 [0.82–1.12]	**0.50 [0.29–0.86]**	**0.51 [0.43–0.61]**	**0.74 [0.62–0.88]**	0.84 [0.30–2.33]
Tertiary	**0.57 [0.42–0.77]**	**0.56 [0.39–0.81]**	**0.57 [0.41–0.79]**	0.97 [0.50–1.88]	**0.29 [0.20–0.42]**	**0.66 [0.51–0.86]**	—
Years of education							
Low (Ref)							
Medium	**0.78 [0.64–0.96]**	**0.8 [0.70–0.93]**	1.11 [0.98–1.25]	—	**0.63 [0.52–0.75]**	**0.83 [0.73–0.94]**	—
High	**0.59 [0.47–0.75]**	**0.64 [0.53–0.76]**	**0.87 [0.75–1.01]**	—	**0.32 [0.25–0.40]**	**0.71 [0.57–0.90]**	—
Wealth							
Low (Ref)							
Medium	**0.72 [0.58–0.88]**	**0.81 [0.71–0.93]**	**0.74 [0.65–0.83]**	1.37 [0.65–2.86]	—	**0.87 [0.77–0.98]**	1.19 [0.80–1.76]
High	**0.62 [0.50–0.78]**	**0.68 [0.60–0.78]**	**0.72 [0.64–0.81]**	0.84 [0.39–1.81]	—	**0.41 [0.36–0.47]**	**0.60 [0.37–0.**96]
Occupation							
Manual (Ref)							
Farmers	—		0.94 [0.78–1.13]			**0.83 [0.73–0.93]**	
Lower nonmanual	0.88 [0.71–1.09]		0.99 [0.83–1.19]			**0.80 [0.71–0.90]**	
Upper nonmanual	**0.73 [0.59–0.91]**		0.74 [0.54–1.00]			**0.71 [0.57–0.89]**	

*Notes*: CI = confidence interval; Ref = reference; SES = socioeconomic status. Results are presented hazard ratios relative to individuals in the reference groups; bold denotes 95% CI does not overlap with 1. Estimates are from Cox Proportion Hazards models with relaxed assumption of the proportionality by allowing the baseline hazard functions to differ by gender.

**Table 4.** Relationship Between Socioeconomic Measures and Mortality, Hazard Ratios (95% CI), Men.

SES Measure	Australia	China	Indonesia	Japan	New Zealand	South Korea	Thailand
Education							
No schooling	—	Ref	Ref	—	—	Ref	Ref
Primary	Ref	**0.84 [0.71–0.99]**	1.18 [1.00–1.39]	Ref	Ref	0.98 [0.81–1.19]	Ref
Secondary	**0.73 [0.57–0.94]**	**0.67 [0.53–0.86]**	1.01 [0.82–1.23]	0.54 [0.28–1.05]	**0.47 [0.37–0.60]**	**0.78 [0.62–0.98]**	0.84 [0.43 – 1.66]
Tertiary	**0.39 [0.26–0.60]**	**0.6 [0.41–0.88]**	**0.5 [0.32–0.78]**	1.05 [0.52–2.12]	**0.27 [0.17–0.45]**	**0.63 [0.46–0.84]**	—
Years of education							
Low (Ref)							
Medium	**0.73 [0.56–0.95]**	**0.84 [0.71–0.99]**	1.18 [1.00–1.40]	—	**0.61 [0.48–0.78]**	**0.83 [0.71–0.97]**	—
High	**0.5 [0.37–0.68]**	**0.66 [0.53–0.83]**	0.89 [0.73–1.08]	—	**0.27 [0.20–0.37]**	**0.63 [0.49–0.81]**	—
Wealth							
Low (Ref)							
Medium	**0.66 [0.50–0.87]**	**0.74 [0.61–0.90]**	**0.71 [0.60–0.85]**	**1.97 [1.04–3.74]**	—	0.9 [0.75–1.09]	1.28 [0.77–2.13]
High	**0.47 [0.35–0.62]**	**0.62 [0.52–0.73]**	**0.7 [0.59–0.84]**	1.16 [0.56–2.42]	—	**0.41 [0.34–0.49]**	**0.46 [0.24–0.87]**
Occupation							
Manual (Ref)							
Farmers	—		0.85 [0.68–1.07]			0.84 [0.71–1.00]	
Lower nonmanual	0.86 [0.63–1.17]		0.85 [0.68–1.07]			**0.69 [0.57–0.84]**	
Upper nonmanual	**0.73 [0.55–0.97]**		**0.68 [0.47–0.98]**			**0.66 [0.51–0.84]**	

*Notes*: CI = confidence interval; Ref = reference; SES = socioeconomic status. Results are presented hazard ratios relative to individuals in the reference groups; bold denotes 95% CI does not overlap with 1. Estimates are from Cox Proportion Hazards models, stratified by gender.

As a robustness check, we also examined the gradients by tertiles of schooling attainment in countries where the number of years of schooling was available (all countries, except for Thailand), with the results presented in Tables 3 and 4. Overall, this relative measure of educational attainment showed very similar patterns to the categorical schooling measure, although the relative gradients in mortality hazard were less steep.

### Wealth

Results exploring inequalities in the hazard of mortality by wealth are presented in Tables 3–5. As shown in Table 3, there was a strong, consistent gradient in later-life mortality risk by wealth. The magnitude of these gradients was substantial—in all countries included (except Japan), the wealthiest third of the population had more than a 30% lower hazard of mortality, and in South Korea, mortality hazard of the wealthiest tertile was less than half of that of the poorest tertile. The gradient in mortality by wealth was relatively weaker in Japan and Thailand, where only the wealthiest third of the population had considerably lower hazards of mortality compared to the poorest. In these two countries, medium levels of household wealth did not seem to confer mortality advantage.

**Table 5.** Relationship Between Socioeconomic Measures and Mortality, Hazard Ratios (95% CI), Women

SES Measure	Australia	China	Indonesia	Japan	New Zealand	South Korea	Thailand
Education							
No schooling	—	Ref	Ref	—	—	Ref	Ref
Primary	Ref	**0.73 [0.55–0.96]**	1.02 [0.85–1.22]	Ref	Ref	0.87 [0.75–1.00]	Ref
Secondary	**0.66 [0.45–0.95]**	**0.6 [0.45–0.80]**	0.89 [0.69–1.15]	0.43 [0.17–1.09]	**0.56 [0.44–0.71]**	**0.72 [0.53–0.98]**	0.96 [0.29–3.23]
Tertiary	0.94 [0.63–1.40]	0.43 [0.14–1.33]	0.75 [0.46–1.24]	0.64 [0.08–5.33]	**0.32 [0.18–0.56]**	1.19 [0.67–2.13]	—
Years of education							
Low (Ref)							
Medium	**0.65 [0.45–0.94]**	**0.73 [0.55–0.96]**	1.02 [0.85–1.22]	—	**0.64 [0.49–0.84]**	0.83 [0.66–1.03]	—
High	0.74 [0.52–1.05]	**0.58 [0.44–0.77]**	0.86 [0.68–1.10]	—	**0.38 [0.27–0.52]**	1.33 [0.80–2.22]	—
Wealth							
Low (Ref)							
Medium	0.79 [0.59–1.06]	0.91 [0.76–1.10]	**0.76 [0.64–0.91]**	0.9 [0.24–3.39]	—	**0.83 [0.71–0.97]**	1.06 [0.57–1.98]
High	0.91 [0.66–1.26]	**0.78 [0.64–0.95]**	**0.74 [0.62–0.88]**	0.57 [0.14–2.36]	—	**0.43 [0.35–0.53]**	0.89 [0.44–1.79]
Occupation							
Manual (Ref)							
Farmers	—		1.24 [0.87–1.75]			**0.82 [0.69–0.98]**	
Lower nonmanual	0.9 [0.68–1.20]		1.37 [0.97–1.92]			0.9 [0.78–1.05]	
Upper nonmanual	0.72 [0.51–1.03]		0.94 [0.56–1.58]			0.91 [0.57–1.46]	

*Notes*: CI = confidence interval; Ref = reference; SES = socioeconomic status. Results are presented hazard ratios relative to individuals in the reference groups; bold denotes 95% CI does not overlap with 1. Estimates are from Cox Proportion Hazards models, stratified by gender.

We observed similar wealth-mortality relationships by gender in all countries, except for Japan. In Japan, wealth conferred mortality advantages for women but not for men. Stratifying analyses by gender, we observed a positive relationship between wealth and mortality among Japanese men, that is, those with the lowest level of wealth had the lowest hazard of mortality. This pattern was reversed among Japanese women. For women, higher household wealth, especially having the highest level of wealth, was associated with lower mortality risk.

### Occupation

We further compared mortality hazards across occupational groups. We note that measures of occupational status were not available in all countries—only Indonesia, Australia, and South Korea had comparable data on occupational groups. The detailed classifications of which occupations were included in each group are provided in Supplementary Table 4.

We found that a mortality gradient based on occupational class was present in all countries included. However, the magnitude of these differences was substantially different across countries. Overall, the gradient in mortality by occupational group was somewhat weaker in Indonesia, where only upper nonmanual laborers seemed to have a mortality advantage over manual laborers. Similarly in Australia, only the highest status workers had a significant mortality advantage. In South Korea, upper nonmanual workers had nearly a 30% lower hazard of mortality, and farmers and lower nonmanual workers also had an advantage over manual laborers.

Although there were no disenable gender differences in the relationship between occupation and mortality in Australia, we found stronger occupational gradients among men compared to women at all levels in South Korea and Indonesia. In Indonesia, in particular, we observed a strong gradient in mortality by occupational group among men. However, employment in lower nonmanual occupations was associated with higher mortality compared to manual occupations among women.

### Sensitivity Analysis

In sensitivity analysis, we stratified by birth cohorts, with results available in Supplementary Table 10. These analyses examine whether the SES gradient in mortality may have shifted over time, particularly as educational attainment and the extent of wealth stratification may have changed over cohorts. Given the small sample sizes in some surveys, we are not able to estimate SES–mortality relationship for more recent cohorts in some countries, particularly in Japan, Thailand, and New Zealand. In addition, these stratified estimates also come with substantial uncertainty due to small sample sizes. Nonetheless, we are able to discern some potential cohort differences in education–mortality relationship in China, Indonesia, and South Korea. These three countries have undergone rapid educational expansion. In South Korea, education gradients were flat for the oldest cohort born before 1940 and they were steeper in more recent cohorts. Similar patterns can be observed in China, although educational gradients in mortality were not statistically significant in recent cohorts born after 1951. In Indonesia, however, we observe flat education gradients (for those with primary and secondary schooling compared to no schooling) in all cohorts including the most recent cohorts included in the study. In contrast, steep wealth-mortality gradients can be observed in all birth cohorts including the older birth cohorts. Results of these stratified analyses highlight that although there are cohort differences in SES–mortality relationship in some countries, cohort differences do not significantly bias our results.

## Discussion

Across a set of seven Asia-Pacific countries, we found substantial social inequalities in mortality among older adults. Inequalities were present across each of the three measures of social stratification (education, wealth, and occupation) investigated. However, there are important heterogeneities in the extent of differences in mortality risk across contexts and by measures of social stratification. With the exception of Japanese men, we found consistent and pervasive gradients in mortality risk in the three established HICs in the region (Australia, New Zealand, and Japan) by all available measures of social stratification. In keeping with other studies from Japan, patterns of mortality inequality are inconsistent and often reversed in this context (Kagamimori et al., 2009; Tanaka et al., 2019), which may be attributable to long working hours and heavy job demands among higher-SES groups in Japan, especially among Japanese men (Lahelma et al., 2010). However, the smaller sample size of the JSTAR survey and geographically limited sample mean that we are somewhat limited in drawing conclusions from these data.

In contrast to HICs, the patterns of inequality in adult mortality in the MICs and recently transitioned HIC investigated (China, Indonesia, South Korea, and Thailand) vary depending on the measure of social stratification. Although we found rather consistent mortality gradients by levels of household wealth, the relationship between education and mortality is mixed. In these countries, only tertiary education conferred a consistent mortality advantage compared to adults with no schooling. We observed flat gradients for primary schooling in China, Indonesia, South Korea, and Thailand, and the mortality advantage for those with secondary schooling was also not pronounced in Indonesia and Thailand.

Our finding of relative lack of differences in mortality risk between individuals with no schooling and those with primary and secondary schooling in LMICs is consistent with evidence from other low- and middle-income contexts including Mexico, South Africa, and Costa Rica (Avendano et al., 2009; Cutler & Lleras-Muney, 2012; Dow & Rehkopf, 2010; Sudharsanan et al., 2020). Prior research has suggested that the selectivity of the no-schooling group may have resulted in flatter gradients. However, we find similar patterns when using a relative measure of schooling, and when we combined the groups of no schooling and primary schooling as the reference group. This suggests that our findings are unlikely to be the result of the selectivity of the reference education groups.

Our findings of the inconsistent relationship between education and mortality and the clear, consistent relationship between household wealth and mortality in the LMICs, may suggest that, among older adults in these contexts, wealth accumulated at a household level is a better indicator of health-promoting resources compared to individual education attainment. It is likely that education, completed at younger ages, may not be a good proxy for resources accumulated through individual life course for older adults in low- and middle-income contexts. Further, despite efforts to strengthen essential health services in recent decades, health systems in a number of LMICs in the Asia-Pacific are characterized by limited coverage of basic health services and high out-of-pocket costs (Tangcharoensathien et al., 2011). With limited social protections, families may act as key providers of income support. As such, household wealth may play an outsized role in its members’ health and well-being, as it is directly linked with one’s ability to afford necessary health care.

We found a mortality gradient by occupational class in the three countries with comparable data on occupational groups. However, in contrast with the consistent wealth-based mortality gradients, mortality differences by occupational groups varied considerably by contexts. Similar to the patterns observed in a number of European countries including England/Wales, France, and Denmark (Tanaka et al., 2019), we found a clear mortality gradient by occupational class in Australia. Previous studies in South Korea have often found higher mortality among nonmanual workers compared to manual workers among the working population (Moatsos et al., 2014; Son et al., 2002; Tanaka et al., 2019). However, focusing on older population groups in South Korea, our analysis showed a mortality advantage among nonmanual workers compared to manual workers. This may suggest considerable cohort differences in the patterns of occupation-based mortality inequalities in South Korea. In contrast to Australia and South Korea, occupational class-based mortality differences were much less pronounced in Indonesia, the only LMIC country with comparable data on occupational groups. In Indonesia, strong wealth-based mortality inequality and relative lack of mortality by education and occupation potentially highlight the importance of accumulated household wealth as a social determinant of health.

We investigated the potential role of health behaviors in differentially influencing the magnitude and direction of the association between SES and mortality in LMICs as compared to HICs. In most HICs, education, wealth, and occupational status are inversely related to poor health-related behaviors (such as smoking, unhealthy consumption of alcohol, and obesity); in contrast, there is some evidence to suggest that SES is positively related to unhealthy level of risk factors in some LMICs (Sudharsanan et al., 2020). Our analyses, however, provide no evidence of systemic differences in SES patterning of health behaviors in LMICs and HICs in the Asia-Pacific, suggesting that behavioral explanations are unlikely to fully explain the observed variations in SES gradients in mortality. Rather, we observe consistent negative SES gradients in smoking in all countries investigated irrespective of their level of development. At the same time, we find that some negative health behaviors are more prevalent among higher-SES individuals in both high-income (i.e., frequent drinking in Australia), as well as middle- and low-income contexts (i.e., overweight/obese BMI in China and Indonesia).

Indeed, although some portion of the relationship between adult SES and mortality undoubtedly operates through health behaviors and health conditions, there is also considerable evidence that an individual’s accumulated exposure to low SES conditions can also have direct impacts on morbidity and mortality. Lower-SES individuals are disproportionately subject to stressors including job insecurity, financial strain, and lack of control over work hours, all of which can have negative health repercussions (Marmot et al., 1998; Schneider & Harknett, 2019). In the LMICs with high out-of-pocket payments, poorer individuals are more likely to face catastrophic health expenditure and are more likely to forfeit necessary health care (Tangcharoensathien et al., 2011). In addition, less-advantaged individuals may have less robust networks of social support, which can act to buffer these stress exposures (Marmot et al., 1998).

Existing studies on HICs have found that national level of income and development are related to differences in countries’ overall levels of mortality and population health, but they are not linked to cross-national variations in the magnitude of SES gradients in mortality (Semyonov et al., 2013). Similarly, we found that the differences in the magnitude of gradients in mortality by wealth and occupation were not associated with a country’s level of national income and development. To illustrate, compared to individuals in the lowest tertile of household wealth, the highest level of household wealth is associated with a similar reduction in mortality risks in Thailand, Australia, and China, countries with drastically different life expectancies, level of income, and development. At the same time, we find that the education gradients in mortality were more pronounced in HICs (Australia and New Zealand) than in lower–middle-income countries (Indonesia and Thailand), with South Korea and China, falling somewhere between. These patterns may suggest that there are substantial differences in the meaning of education across contexts. Perhaps, apart from having tertiary education, education is not as indicative of an individual’s socioeconomic standing in LMICs compared to HICs in the Asia-Pacific.

In common with other research investigating cross-national patterns, our analyses are not without limitations. Our thresholds of schooling attainment and occupational classification may represent different levels of attained SES across countries—that is, completing tertiary education is likely a much stronger marker of attained SES in Indonesia than in Australia. In measuring household wealth, our analyses do not account for whether the respondent has access to or agency over the household’s wealth. Our results are also observational, and as such, we do not comprehensively investigate potential determinants of SES-based mortality disparities. Loss-to-follow-up rates varied substantially between countries, and although we use IPW to adjust for potential bias on observable characteristics there may be other unadjusted, or unobserved, factors related to attrition. The source data on mortality also varied somewhat between the studies used, and aside from the HILDA (Australia) and NZHWR surveys, were based on survey follow-up rather than linkages to national death records. This variation in linkage to death records means that our analyses are unable to compare absolute levels of mortality across contexts, as these could be subject to bias due to country-level differences in how mortality data were collected. Finally, our findings are for seven specific countries in the Asia-Pacific region, and as such they may not generalize to other countries in the region.

Social inequalities in mortality are pervasive across the world, present in contexts ranging from high-income, relatively egalitarian Nordic countries (Katikireddi et al., 2020; Rognerud & Zahl, 2006) to subsistence societies (Jaeggi et al., 2021). Analyzing these gradients in the Asia-Pacific shows that inequalities in later-life health are present across the region, and that social gradients in mortality are overall larger in HICs as compared to MICs. In addition, we find that social inequalities in mortality do not closely align with social inequalities in health behaviors. A combination of factors may be driving these patterns, including psychosocial stress among lower-SES individuals and unequal access to and quality of health care (Kruk et al., 2018). Our findings highlight that future research is needed to further understand the mechanisms underlying mortality inequalities across contexts.

## Supplementary Material

gbad193_suppl_Supplementary_Tables_S1-S10
