# Supplementary material for: Inequalities in Mortality in the Asia-Pacific: A Cross-National Comparison of Socioeconomic Gradients
Source: J Gerontol B Psychol Sci Soc Sci. 2024 Jan 9;79(4):gbad193. doi: 10.1093/geronb/gbad193 (PMC10948962; doi:10.1093/geronb/gbad193)
Supplement: gbad193_suppl_Supplementary_Tables_S1-S10 [file gbad193_suppl_supplementary_tables_s1-s10.docx]

Supplementary Table 1: Sampling information for each survey

| Survey name | Years of data used in study | Eligibility criteria | Sample design | Mortality assessment |
| --- | --- | --- | --- | --- |
| Health, Income and Labour Dynamics in Australia | 2000-2018 annual surveys | All households | First stage: random sample of census collection districts  Second stage: random sample of dwellings  Third stage: random sample of HHs  Interviews: HH member with most knowledge of HH finances and any HH member aged 15+ | Sample matched with National Death Index for date of death |
| China Health and Retirement Longitudinal Study | 2011, 2013, 2015, 2018 | Households with members aged 45+ | First stage: random sample of counties  Second stage: random sample of PSUs  Third stage: random sample of HHs  Interviews: a randomly selected individual aged 45+ and their spouse | Household or family-member reported date of death at each follow-up wave |
| Indonesian Family Life Survey | 2007, 2014/15 | All households | First stage: random sample of census enumeration areas  Second stage: random sample of HH’s  Interviews: HH head and spouse, two randomly selected children, a randomly selected individual aged 50+ and their spouse. | Household-member reported date of death at each follow-up wave |
| Japanese Study of Aging and Retirement | 2007, 2009, 2011, 2013 | Individuals aged 50+ | First stage: Non-random selection of municipalities  Second stage: random sample of locations determined by household registry  Third stage: Random sample of HHs  Interviews: One person per household aged 50+ | Household-member reported date of death at each follow-up wave |
| New Zealand Health, Work and Retirement Survey | 2008, 2010 | Individuals aged 55+ | Sub sample one: random sample of individuals aged 55+ from Electoral Roll (adults aged 18+)  Sub sample two: random sample of Maori (Indigenous) individuals aged 55+ from Electoral Roll | Sample matched with national health and mortality records for date of death |
| Korean Longitudinal Study on Ageing | 2006, 2008, 2010, 2012, 2014, 2016, 2018 | Individuals aged 45+ | First stage: random sample of enumeration districts  Second stage: random sample of HH’s with member aged 45+  Interviews:  Strata: urban/rural, apartment/house | Household-member reported date of death at each follow-up wave |
| Health, Aging and Retirement in Thailand | 2015, 2016 | Households with members aged 45+ | First stage: stratified random sample of provinces  Second stage: sample from capital of province and stratified random sample of other district  Third stage: random sample of rural village and/or urban block  Fourth stage: random sample of HHs with member aged 45+  Interviews: One randomly selected individual aged 45+ | Household-member reported date of death at follow-up wave |

Supplementary Table 2: Classification of country-specific educational attainment categories

| Survey name | None | Primary | Secondary | Tertiary |
| --- | --- | --- | --- | --- |
| Health, Income and Labour Dynamics in Australia | • Attended primary school but did not finish | • Primary school | • High school  • Certificate I-IV  • Diploma  • Advanced diploma | • Associate Degree  • Bachelor Degree  • Grad diploma and Grad Certificate  • Bachelor (Honours) Degree  • Master Degree  • Doctoral Degree  • Postgraduate |
| China Health and Retirement Longitudinal Study | • No formal education  • Did not finish primary school  • Sishu | • Elementary school | • Middle school  • High school  • Vocational school | • Two/three year college/Associate degree  • Four year college/Bachelor’s degree  •Post-graduated (Master/PhD) |
| Indonesian Family Life Survey | • None | • Elementary school  • Adult education A  • Islamic Elementary school | • Junior high - general  • Junior high - vocational  • Senior high - general  • Senior high - vocational  • Adult education C  • Islamic Junior high school  • Islamic Senior high school | • Open University  • College D1, D2, D3  • University S1  • University S2  • University S3 |
| Japanese Study of Aging and Retirement |  | • Elementary/middle school | • High school (Including old-system middle school, girls’ school, trade school, normal school)  • Junior college (Including technical high school, etc.)  • Vocational school | • University (Including old-system high school, old-system technical college)  • Graduate school (Master’s)  • Graduate school (Ph.D.) |
| New Zealand Health, Work and Retirement Survey |  | • No school qualifications | • Secondary school  • Post-secondary/trade | • Tertiary |
| Korean Longitudinal Study on Ageing | • No education (illiterate)  • No education (reading) | • Elementary school | • Middle school  • High school | • Two-year grad  • College grad  • Post college (Master)  • Post college (PhD) |
| Health, Aging and Retirement in Thailand | • No formal education (illiterate)  • No formal education (literate) | • Elementary school | • Middle school  • High school  • Vocational Diploma (two-year diploma degree) | • Bachelor degree  • Higher than bachelor degree |

Supplementary Table 3: Variables used to generate household wealth

| Survey name | Financial wealth | Durable wealth | Non-financial wealth | Debts |
| --- | --- | --- | --- | --- |
| Health, Income and Labour Dynamics in Australia | • Bank accounts  • Superannuation  • Cash and equity investments  • Trust funds  • Life insurance  • Business | • Collectables and other assets  • Vehicles | • Primary residence  • Other real estate | • Credit card debt  • Higher Education Contribution Scheme debt  • Business debt  • Other loans  • Mortgage |
| China Health and Retirement Longitudinal Study | • Cash, checking and saving accounts  • Stocks and mutual funds  • Government bonds  • All other savings  • Personal loans lent | • Vehicles  • Consumer durable assets*  • Fixed capital assets^#^ | • Primary residence  • Other real estate  • Irrigable land  • Livestock and fisheries | • Mortgage  • Other debt |
| Indonesian Family Life Survey | •Savings, certificates of deposit or stocks  • Household receivables | • Vehicles  • Consumer durable assets^?^  • Other assets  • Assets from non-farm business^a^  • Assets from farm business^b^ | • Primary residence  • Poultry  • Livestock/fish pond  • Hard stem plants | • Loans |
| Japanese Study of Aging and Retirement | • Savings  • Bonds  • Stocks  • Business | N/A | • Primary residence  • Other real estate | • Mortgage  • Other debt |
| Korean Longitudinal Study on Ageing | • Business  • Stocks and mutual funds  • Cash, checking and saving accounts  • Bonds and bond funds  • Other savings and assets  • Rental safety deposit paid  • Other security deposit paid | • Vehicle  • Other assets | • Primary residence  • Other real estate | • Mortgage  • Rental safety deposit received  • Other security deposit received  • Other debt |
| Health, Aging and Retirement in Thailand | • Business  • Cash and checking accounts  • Deposits and savings  • Stocks and mutual funds  • Government bonds  • Saving cooperative  • Insurance settlement  • Other financial assets | • Vehicle or boat  • Other assets | • Primary residence  • Other real estate | • Mortgage  • Saving cooperative debt  • Financial institution/credit card loan  • Personal loans from friends  • Other debt |

*Consumer durable assets includes refrigerator, washing machine, TV, computer, stereo system, camera, air conditioner, mobile phone, furniture, music instrument, valuable decorations, ornaments, and vases, treasures and precious metals, antiques

# Fixed capital assets include tractor, thresher, tractor tools, water pump, processing equipment, current value of fixed capital used in household production of self-employment activities, value of other durable or fixed assets worth 500 yuan and more

? Consumer durable assets includes household appliances, jewelry, furniture and utensils

^a^ Non-farm business assets includes land, building, four-wheel motor vehicles, other vehicles, other non-farm equipment

^a^ Farm business assets includes farm land, poultry, livestock/fish pond, hard stem plants, house or building used for the farm business, vehicles, tractor, irrigation equipment, heavy equipment, small tools, fishing equipment, other assets

Supplementary Table 4: Classification of occupation groups

| Survey name | Farmer | Manual worker | Lower non-manual | Upper non-manual |
| --- | --- | --- | --- | --- |
| Health, Income and Labour Dynamics in Australia | • Laborers and related workers | • Tradesperson and related workers  • Intermediate production and transport workers | • Elementary clerical, sales and service workers  • Intermediate clerical, sales and services workers  • Advanced clerical and service workers | • Managers and administrators  • Professionals  • Associate professionals |
| China Health and Retirement Longitudinal Study |  |  |  |  |
| Indonesian Family Life Survey | • Agricultural workers | • Production and related workers, transport operators and labourers | • Clerical and related workers  • Sales workers  • Service workers  • Military and police | • Professional or technical  • Managerial or government administrator |
| Japanese Study of Aging and Retirement |  |  |  |  |
| New Zealand Health, Work and Retirement Survey |  |  |  |  |
| Korean Longitudinal Study on Ageing | • Agricultural, forestry and fishery worker | • Craftsman, mining worker, manufacturing and construction worker, labourer  • Worker in transport and communication  Protective service worker | • Clerical and related worker  • Sales worker  • Service worker | • Manager and official  • Professional and technical worker |
| Health, Aging and Retirement in Thailand |  |  |  |  |

Supplementary Table 5: Age at death distribution

| Age and death distribution, Australia | | | | |
| --- | --- | --- | --- | --- |
| Age group | Men |  | Women |  |
|  | Individuals | Deaths | Individuals | Deaths |
| 45 | 761 | 14 | 832 | 6 |
| 50 | 825 | 17 | 876 | 10 |
| 55 | 692 | 19 | 804 | 8 |
| 60 | 622 | 14 | 673 | 10 |
| 65 | 563 | 36 | 621 | 14 |
| 70 | 428 | 38 | 475 | 27 |
| 75 | 332 | 61 | 395 | 39 |
| 80 | 288 | 83 | 315 | 57 |
| 85+ | 243 | 90 | 406 | 143 |
| Total | 4,754 | 372 | 5,397 | 314 |

| Age and death distribution, China | | | | |
| --- | --- | --- | --- | --- |
| Age group | Men |  | Women |  |
|  | Individuals | Deaths | Individuals | Deaths |
| 45 | 848 | 29 | 1,240 | 14 |
| 50 | 2,014 | 67 | 2,150 | 45 |
| 55 | 1,768 | 93 | 1,940 | 46 |
| 60 | 2,050 | 153 | 2,033 | 90 |
| 65 | 1,845 | 193 | 1,869 | 81 |
| 70 | 1,270 | 181 | 1,223 | 119 |
| 75 | 955 | 218 | 894 | 146 |
| 80 | 593 | 168 | 577 | 130 |
| 85+ | 325 | 119 | 438 | 176 |
| Total | 11,668 | 1,221 | 12,364 | 847 |

| Age and death distribution, Indonesia | | | | |
| --- | --- | --- | --- | --- |
| Age group | Men |  | Women |  |
|  | Individuals | Deaths | Individuals | Deaths |
| 45 | 1,529 | 47 | 1,513 | 52 |
| 50 | 1,225 | 67 | 1,347 | 57 |
| 55 | 1,009 | 100 | 1,077 | 67 |
| 60 | 819 | 93 | 883 | 88 |
| 65 | 568 | 115 | 639 | 129 |
| 70 | 523 | 144 | 644 | 138 |
| 75 | 333 | 116 | 415 | 123 |
| 80 | 205 | 85 | 302 | 126 |
| 85+ | 191 | 88 | 259 | 114 |
| Total | 6,402 | 855 | 7,079 | 894 |

| Age and death distribution, Japan | | | | |
| --- | --- | --- | --- | --- |
| Age group | Men |  | Women |  |
|  | Individuals | Deaths | Individuals | Deaths |
| 50 | 201 | 2 | 239 | 1 |
| 55 | 511 | 5 | 587 | 2 |
| 60 | 682 | 15 | 710 | 5 |
| 65 | 685 | 18 | 708 | 6 |
| 70 | 686 | 38 | 753 | 13 |
| 75 | 428 | 20 | 454 | 15 |
| 80 | 81 | 6 | 86 | 4 |
| 85+ | 1 | 1 | 1 | 1 |
| Total | 3,275 | 105 | 3,538 | 47 |

| Age and death distribution, New Zealand | | | | |
| --- | --- | --- | --- | --- |
| Age group | Men |  | Women |  |
|  | Individuals | Deaths | Individuals | Deaths |
| 50 | 113 | 1 | 127 | 0 |
| 55 | 676 | 18 | 805 | 27 |
| 60 | 742 | 90 | 886 | 75 |
| 65 | 803 | 124 | 971 | 111 |
| 70 | 406 | 100 | 472 | 97 |
| 75 | 188 | 28 | 203 | 30 |
| 80+ | 2 | 2 | -- | -- |
| Total | 2930 | 363 | 3464 | 340 |

| Age and death distribution, South Korea | | | | |
| --- | --- | --- | --- | --- |
| Age group | Men |  | Women |  |
|  | Individuals | Deaths | Individuals | Deaths |
| 45 | 144 | 5 | 174 | 2 |
| 50 | 227 | 34 | 266 | 21 |
| 55 | 797 | 63 | 1,026 | 37 |
| 60 | 709 | 72 | 831 | 61 |
| 65 | 668 | 121 | 770 | 60 |
| 70 | 686 | 158 | 760 | 103 |
| 75 | 676 | 215 | 874 | 185 |
| 80 | 583 | 226 | 743 | 221 |
| 85+ | 405 | 215 | 830 | 416 |
| Total | 4,895 | 1,109 | 6,274 | 1,106 |

| Age and death distribution, Thailand | | | | |
| --- | --- | --- | --- | --- |
| Age group | Men |  | Women |  |
|  | Individuals | Deaths | Individuals | Deaths |
| 45 | 72 | 2 | 91 | 1 |
| 50 | 161 | 3 | 190 | 5 |
| 55 | 200 | 0 | 227 | 2 |
| 60 | 227 | 8 | 276 | 2 |
| 65 | 274 | 10 | 295 | 1 |
| 70 | 212 | 5 | 234 | 8 |
| 75 | 227 | 10 | 232 | 6 |
| 80 | 190 | 14 | 218 | 15 |
| 85+ | 184 | 26 | 232 | 14 |
| Total | 1,747 | 78 | 1,995 | 54 |

Supplementary Table 6: Country descriptors

| Country | Years of survey data included | 2005 N’000 adults aged 50+  (% of total N)^a^ | 2020 N’000 adults aged 50+  (% of total N) | 2040 N’000 adults aged 50+  (% of total N)^b^ | World Bank Income Group^c^ |
| --- | --- | --- | --- | --- | --- |
| Australia | 2006, 2010, 2014 | 6,078 (30%) | 8,661 (35%) | 11,954 (39%) | HI |
| China | 2011, 2013, 2015, 2018 | 289, 270 (22%) | 471,962 (33%) | 654,245 (45%) | UMI |
| Indonesia | 2007, 2014 | 33,358 (15%) | 56,679 (21%) | 95,523 (30%) | LMI |
| Japan | 2007, 2009, 2011, 2013 | 53,103 (41%) | 59,954 (47%) | 62,256 (55%) | HI |
| New Zealand | 2006,2008, 2010,2012,2014 | 1,171 (28%) | 1,700 (35%) | 2,167 (39%) | HI |
| South Korea | 2006, 2008, 2010, 2012, 2014, 2016, 2018 | 11,513 (24%) | 20,347 (40%) | 27,088 (54%) | HI |
| Thailand | 2015, 2017 | 14,125 (22%) | 24,094 (35%) | 31,550 (46%) | UMI |

^Notes: HI = High Income, UMI = Upper middle Income, LMI = Lower Middle Income^

^a^https://population.un.org/wpp/Download/Standard/Population/

^b^ https://population.un.org/wpp/Download/Probabilistic/Population/

^c^<https://datahelpdesk.worldbank.org/knowledgebase/articles/906519-world-bank-country-and-lending-groups>

Supplementary Table 7: Age-standardized probability of ever smoking by measures of socioeconomic status [95% CI]

| **Variable** | **Australia** | **China** | **Indonesia** | **Japan** | **New Zealand*** | **South Korea** | **Thailand** |
| --- | --- | --- | --- | --- | --- | --- | --- |
| **Male** |  |  |  |  |  |  |  |
| **Education** |  |  |  |  |  |  |  |
| No schooling | -- | 0.78 [0.76 - 0.79] | 0.80 [0.79 - 0.82] | -- | 0.22 [0.20 - 0.25] | 0.72 [0.68 - 0.76] | 0.63 [0.49 - 0.78] |
| Primary | 0.67 [0.65 - 0.69] | 0.73 [0.72 - 0.75] | 0.76 [0.75 - 0.78] | 0.80 [0.78 - 0.81] | 0.17 [0.15 - 0.19] | 0.66 [0.64 - 0.68] | 0.64 [0.62 - 0.67] |
| Secondary | 0.64 [0.62 - 0.66] | 0.71 [0.70 - 0.73] | 0.72 [0.70 - 0.74] | 0.78 [0.75 - 0.82] | 0.13 [0.11 - 0.15] | 0.61 [0.59 - 0.63] | 0.54 [0.49 - 0.59] |
| Tertiary | 0.52 [0.50 - 0.55] | 0.60 [0.56 - 0.65] | 0.60 [0.56 - 0.64] | 0.75 [0.72 - 0.77] | 0.07 [0.05 - 0.09] | 0.51 [0.48 - 0.54] | -- |
| **Wealth** |  |  |  |  |  |  |  |
| Low | 0.73 [0.71 - 0.75] | 0.77 [0.76 - 0.78] | 0.81 [0.79 - 0.82] | 0.83 [0.81 - 0.84] | -- | 0.68 [0.66 - 0.71] | 0.65 [0.61 - 0.68] |
| Medium | 0.61 [0.59 - 0.63] | 0.73 [0.72 - 0.74] | 0.77 [0.75 - 0.79] | 0.77 [0.75 - 0.79] | -- | 0.63 [0.61 - 0.66] | 0.64 [0.62 - 0.67] |
| High | 0.53 [0.51 - 0.55] | 0.70 [0.69 - 0.71] | 0.68 [0.66 - 0.70] | 0.75 [0.73 - 0.78] | -- | 0.57 [0.55 -0.59] | 0.59 [0.56 - 0.63] |
| **Occupation** |  |  |  |  |  |  |  |
| Manual (ref) | 0.66 [0.64 - 0.68] | -- | 0.78 [0.76 - 0.80] | -- | -- | 0.52 [0.49 - 0.56] | -- |
| Farmers | -- |  | 0.79 [0.77 - 0.81] |  |  | 0.62 [0.59 -0.64] |  |
| Lower non-manual | 0.65 [0.63 - 0.67] |  | 0.74 [0.72 - 0.76] |  |  | 0.66 [0.64 -0.69] |  |
| Upper non-manual | 0.59 [0.57 - 0.61] |  | 0.62 [0.59 - 0.66] |  |  | 0.63 [0.59 -0.66] |  |
| **Female** |  |  |  |  |  |  |  |
| **Education** |  |  |  |  |  |  |  |
| No schooling | -- | 0.08 [0.08 - 0.09] | 0.05 [0.05 - 0.06] | -- | 0.24 [0.22 -0.26] | 0.05 [0.04 - 0.06] | 0.06 [0.03 - 0.10] |
| Primary | 0.51 [0.49 - 0.53] | 0.07 [0.06 - 0.07] | 0.04 [0.04 - 0.05] | 0.17 [0.16 - 0.19] | 0.18 [0.16 - 0.21] | 0.04 [0.03 - 0.04] | 0.07 [0.05 - 0.08] |
| Secondary | 0.48 [0.46 - 0.50] | 0.06 [0.06 - 0.07] | 0.03 [0.03 - 0.04] | 0.16 [0.14 - 0.19] | 0.14 [0.12 - 0.16] | 0.03 [0.03 - 0.03] | 0.04 [0.03 - 0.06] |
| Tertiary | 0.36 [0.34 - 0.38] | 0.04 [0.03 - 0.05] | 0.02 [0.02 - 0.02] | 0.14 [0.12 - 0.16] | 0.07 [0.05 - 0.09] | 0.02 [0.02 - 0.02] | -- |
| **Wealth** |  |  |  |  |  |  |  |
| Low | 0.58 [0.56 - 0.60] | 0.09 [0.08 - 0.10] | 0.06 [0.05 -0.07] | 0.21 [0.19 - 0.22] | -- | 0.04 [0.04 - 0.05] | 0.07 [0.05 - 0.08] |
| Medium | 0.45 [0.43 - 0.47] | 0.07 [0.07 - 0.08] | 0.05 [0.04 - 0.05] | 0.15 [0.14 - 0.17] | -- | 0.03 [0.03 - 0.04] | 0.07 [0.05 - 0.08] |
| High | 0.36 [0.34 - 0.38] | 0.06 [0.06 - 0.07] | 0.03 [0.03 -0.03] | 0.14 [0.13 - 0.16] | -- | 0.03 [0.02 - 0.03] | 0.05 [0.04 - 0.07] |
| **Occupation** |  |  |  |  |  |  |  |
| Manual (ref) | 0.50 [0.48 - 0.52] | -- | 0.05 [0.04 - 0.06] | -- | -- | 0.02 [0.02 - 0.03] | -- |
| Farmers | -- |  | 0.06 [0.05 - 0.06] |  |  | 0.03 [0.03 - 0.04] |  |
| Lower non-manual | 0.49 [0.47 - 0.51] |  | 0.04 [0.04 - 0.05] |  |  | 0.04 [0.04 - 0.05] |  |
| Upper non-manual | 0.43 [0.41 - 0.45] |  | 0.03 [0.02 - 0.03] |  |  | 0.04 [0.03 - 0.04] |  |

Notes: NZHWR (New Zealand) asked only about current smoking; these figures are not directly comparable to other countries

Supplementary Table 8: Age-standardized probability of frequent drinking (drinking >4 days a week) by measures of socioeconomic status [95 CI]

| **Variable** | **Australia** | **China** | **Indonesia** | **Japan** | **New Zealand** | **South Korea** | **Thailand** |
| --- | --- | --- | --- | --- | --- | --- | --- |
| **Male** |  |  |  |  |  |  |  |
| **Education** |  |  |  |  |  |  |  |
| No formal schooling | -- | 0.26 [0.25 - 0.28] | -- | -- | 0.22 [0.20 - 0.24] | 0.15 [0.12 - 0.19] | 0.14 [0.00 - 0.30] |
| Primary schooling | 0.39 [0.37 - 0.41] | 0.26 [0.24 - 0.27] | -- | 0.50 [0.48 - 0.52] | 0.33 [0.31 - 0.36] | 0.15 [0.13 - 0.17] | 0.09 [0.08 - 0.11] |
| Secondary schooling | 0.47 [0.45 - 0.49] | 0.23 [0.22 - 0.25] | -- | 0.54 [0.49 - 0.59] | 0.34 [0.31 - 0.37] | 0.11 [0.09 - 0.12] | 0.08 [0.05 - 0.11] |
| Tertiary | 0.52 [0.50 - 0.55] | 0.15 [0.11 - 0.19] | -- | 0.49 [0.45 - 0.52] | 0.43 [0.39 - 0.48] | 0.07 [0.05 - 0.09] | -- |
| **Wealth** |  |  |  |  |  |  |  |
| Low | 0.37 [0.35 - 0.38] | 0.26 [0.24 - 0.27] | -- | 0.47 [0.44 - 0.49] | -- | 0.13 [0.11 -0.15] | 0.12 [0.09 - 0.14] |
| Medium | 0.44 [0.42 - 0.46] | 0.25 [0.24 - 0.26] | -- | 0.51 [0.48 - 0.54] | -- | 0.13 [0.11 - 0.14] | 0.07 [0.05 - 0.09] |
| High | 0.57 [0.55 - 0.59] | 0.24 [0.22 - 0.25] | -- | 0.51 [0.49 - 0.54] | -- | 0.10 [0.09 -0.12] | 0.08 [0.06 - 0.10] |
| **Occupation** |  |  |  |  |  |  |  |
| Manual | 0.33 [0.31 - 0.35] |  | -- | -- | -- | 0.08 [0.07 - 0.10] | -- |
| Farmers | -- |  |  |  |  | 0.11 [0.09 - 0.13] |  |
| Lower non-manual | 0.47 [0.45 - 0.49] |  |  |  |  | 0.13 [0.12 - 0.14] |  |
| Upper non-manual | 0.52 [0.50 - 0.54] |  |  |  |  | 0.15 [0.12 - 0.17] |  |
| **Female** |  |  |  |  |  |  |  |
| **Education** |  |  |  |  |  |  |  |
| No formal schooling | -- | 0.02 [0.01 - 0.02] | -- | -- | 0.12 [0.10 -0.13] | 0.01 [0.01 -0.01] | 0.01 [0.00 - 0.02] |
| Primary schooling | 0.23 [0.22 - 0.24] | 0.02 [0.02 - 0.02] | -- | 0.13 [0.12 - 0.14] | 0.19 [0.17 - 0.22] | 0.01 [0.01 - 0.01] | 0.01 [0.00 - 0.01] |
| Secondary schooling | 0.30 [0.28 - 0.31] | 0.02 [0.01 - 0.02] | -- | 0.15 [0.13 - 0.17] | 0.20 [0.18 - 0.22] | 0.01 [0.00 - 0.01] | 0.00 [0.00 - 0.01] |
| Tertiary | 0.34 [0.32 - 0.36] | 0.01 [0.01 - 0.01] | -- | 0.12 [0.10 - 0.14] | 0.27 [0.23 - 0.30] | 0.00 [0.00 - 0.01] | -- |
| **Wealth** |  |  |  |  |  |  |  |
| Low | 0.21 [0.19 - 0.22] | 0.02 [0.02 - 0.02] | -- | 0.12 [0.10 - 0.13] | -- | 0.01 [0.01 -0.01] | 0.01 [0.00 - 0.01] |
| Medium | 0.26 [0.24 - 0.28] | 0.02 [0.02 - 0.02] | -- | 0.14 [0.12 - 0.15] | -- | 0.01 [0.01 - 0.01] | 0.00 [0.00 - 0.01] |
| High | 0.37 [0.35 - 0.39] | 0.02 [0.01 - 0.02] | -- | 0.14 [0.13 - 0.16] | -- | 0.01 [0.00 - 0.01] | 0.01 [0.00 - 0.01] |
| **Occupation** |  |  |  |  |  |  |  |
| Manual | 0.18 [0.17 - 0.20] | -- | -- | -- | -- | 0.01 [0.00 - 0.00] | -- |
| Farmers | -- |  |  |  |  | 0.01 [0.01 - 0.01] |  |
| Lower non-manual | 0.29 [0.27 - 0.30] |  |  |  |  | 0.01 [0.01 - 0.01] |  |
| Upper non-manual | 0.33 [0.31 - 0.34] |  |  |  |  | 0.01 [0.01 - 0.01] |  |

Notes: NZHWR (New Zealand) asked only about current smoking; these figures are not directly comparable to other countries

Supplementary Table 8: Age-standardized probability of frequent drinking (drinking >4 days a week) by measures of socioeconomic status [95 CI]

Supplemental Table 9: Age-standardized probability of overweight/obese body mass index (BMI>25) by measures of socioeconomic status [95 CI]

| **Variable** | **Australia** | **China** | **Indonesia** | **Japan** | **New Zealand** | **South Korea** | **Thailand** |
| --- | --- | --- | --- | --- | --- | --- | --- |
| **Male** |  |  |  |  |  |  |  |
| **Education** |  |  |  |  |  |  |  |
| No formal schooling | -- | 0.24 [0.22 - 0.25] | 0.15 [0.14 - 0.17] | -- | -- | 0.20 [0.17 - 0.22] | 0.24 [0.16 -0.33] |
| Primary schooling | 0.73 [0.71 - 0.75] | 0.28 [0.27 - 0.29] | 0.22 [0.20 - 0.23] | 0.28 [0.27 - 0.30] |  | 0.25 [0.23 - 0.26] | 0.27 [0.24 - 0.29] |
| Secondary schooling | 0.70 [0.69 - 0.72] | 0.30 [0.29 -0.32] | 0.28 [0.27 - 0.30] | 0.24 [0.20 - 0.27] |  | 0.20 [0.18 - 0.21] | 0.28 [0.24 - 0.32] |
| Tertiary | 0.65 [0.63 - 0.67] | 0.44 [0.38 -0.49] | 0.40 [0.36 - 0.43] | 0.24 [0.21 - 0.27] |  | 0.18 [0.16 - 0.20] |  |
| **Wealth** |  |  |  |  |  |  |  |
| Low | 0.73 [0.71 - 0.75] | 0.25 [0.24 -0.26] | 0.18 [0.17 - 0.19] | 0.30 [0.27 - 0.32] | -- | 0.21 [0.20 - 0.23] | 0.27 [0.24 - 0.30] |
| Medium | 0.72 [0.70 - 0.74] | 0.28 [0.27 - 0.29] | 0.21 [0.20 - 0.23] | 0.26 [0.24 - 0.29] |  | 0.21 [0.19 - 0.22] | 0.26 [0.23 - 0.29] |
| High | 0.65 [0.64 - 0.67] | 0.32 [0.30 - 0.33] | 0.31 [0.29 - 0.32] | 0.25 [0.23 - 0.27] |  | 0.21 [0.20 - 0.23] | 0.28 [0.25 - 0.31] |
| **Occupation** |  |  |  |  |  |  |  |
| Manual | 0.71 [0.70 - 0.73] | -- | 0.20 [0.18 - 0.21] | -- | -- | 0.20 [0.18 - 0.23] | -- |
| Farmers | -- |  | 0.13 [0.12 - 0.14] |  |  | 0.23 [0.21 - 0.25] |  |
| Lower non-manual | 0.70 [0.68 - 0.72] |  | 0.30 [0.28 - 0.31] |  |  | 0.22 [0.20 - 0.23] |  |
| Upper non-manual | 0.69 [0.67 - 0.71] |  | 0.38 [0.35 - 0.41] |  |  | 0.16 [0.14 - 0.19] |  |
| **Female** |  |  |  |  |  |  |  |
| **Education** |  |  |  |  |  |  |  |
| No formal schooling | -- | 0.34 [0.33 - 0.35] | 0.32 [0.30 - 0.33] | -- | -- | 0.21 [0.19 -0.24] | 0.34 [0.24 - 0.44] |
| Primary schooling | 0.61 [0.59 - 0.62] | 0.39 [0.38 - 0.41] | 0.41 [0.39 - 0.43] | 0.22 [0.21 - 0.24] |  | 0.26 [0.25 -0.28] | 0.36 [0.34 -0.39] |
| Secondary schooling | 0.57 [0.55 - 0.59] | 0.42 [0.40 -0.44] | 0.50 [0.48 - 0.52] | 0.18 [0.16 - 0.21] |  | 0.21 [0.19 -0.23] | 0.38 [0.33 -0.43] |
| Tertiary | 0.52 [0.49 - 0.54] | 0.56 [0.50 -0.62] | 0.62 [0.59 - 0.66] | 0.19 [0.16 - 0.21] |  | 0.19 [0.16 - 0.21] | -- |
| **Wealth** |  |  |  |  |  |  |  |
| Low | 0.61 [0.59 - 0.63] | 0.34 [0.32 - 0.35] | 0.32 [0.30 -0.33] | 0.24 [0.22 - 0.26] |  | 0.24 [0.22 - 0.25] | 0.36 [0.33 - 0.39] |
| Medium | 0.59 [0.57 - 0.61] | 0.37 [0.36 - 0.39] | 0.37 [0.35 - 0.39] | 0.21 [0.19 -0.23] |  | 0.23 [0.21 - 0.25] | 0.35 [0.32 - 0.39] |
| High | 0.52 [0.50 - 0.54] | 0.41 [0.40 - 0.43] | 0.48 [0.47 - 0.50] | 0.19 [0.18 - 0.21] |  | 0.24 [0.22 - 0.25] | 0.38 [0.35 - 0.41] |
| **Occupation** |  |  |  |  |  |  |  |
| Manual | 0.59 [0.57 - 0.61] | -- | 0.33 [0.31 - 0.36] | -- | -- | 0.22 [0.19 - 0.25] | -- |
| Farmers | -- |  | 0.23 [0.21 - 0.25] |  |  | 0.25 [0.23 - 0.26] |  |
| Lower non-manual | 0.58 [0.56 - 0.60] |  | 0.46 [0.45 - 0.48] |  |  | 0.24 [0.22 - 0.25] |  |
| Upper non-manual | 0.56 [0.55 - 0.58] |  | 0.55 [0.52 - 0.59] |  |  | 0.18 [0.16 - 0.20] |  |

Supplementary Table 10: Relationship between SES measures and mortality stratified by cohorts, hazard ratios [95% CI]

| **Variable** | **Australia** | **China** | **Indonesia** | **Japan** | **New Zealand** | **South Korea** | **Thailand** |
| --- | --- | --- | --- | --- | --- | --- | --- |
| **Education** |  |  |  |  |  |  |  |
| **(<1940)** |  |  |  |  |  |  |  |
| No schooling | -- | Ref | Ref | -- | -- | Ref | Ref |
| Primary | Ref | 0.84 [0.67 - 1.06] | 1.05 [0.89 - 1.25] | Ref | Ref | 0.90 [0.77 - 1.05] | 0.74 [0.31 - 1.76] |
| Secondary | 0.72 [0.58 - 0.90] | 0.49 [0.33 - 0.72] | 1.06 [0.83 - 1.35] | 0.23 [0.04 - 1.42] | 0.58 [0.44 - 0.77] | 0.82 [0.62 - 1.09] | 1.40 [0.47 - 4.21] |
| Tertiary | 0.79 [0.57 - 1.10] | 0.75 [0.50 - 1.13] | 0.33 [0.15 - 0.74] | 0.00 [0.00 – 0.00] | 0.23 [0.11 - 0.47] | 0.80 [0.55 - 1.16] | -- |
| **(1941-1950)** |  |  |  |  |  |  |  |
| No schooling | -- | Ref | Ref | -- | -- | Ref | -- |
| Primary | Ref | 0.76 [0.61 - 0.95] | 1.13 [0.88 - 1.43] | Ref | Ref | 0.93 [0.77 - 1.14] | -- |
| Secondary | 0.71 [0.38 - 1.33] | 0.59 [0.44 - 0.78] | 0.95 [0.69 - 1.29] | 0.33 [0.05 - 2.44] | 0.51 [0.41 - 0.62] | 0.68 [0.51 - 0.92] | -- |
| Tertiary | 0.22 [0.09 - 0.55] | 0.32 [0.11 - 0.85] | 0.88 [0.53 - 1.47] | 1.75 [0.73 - 4.21] | 0.33 [0.22 - 0.49] | 0.65 [0.45 - 0.95] | -- |
| **(1951-1960)** |  |  |  |  |  |  |  |
| No schooling | -- | Ref | Ref | -- | -- | Ref | -- |
| Primary | Ref | 0.83 [0.59 - 1.17] | 1.26 [0.96 - 1.66] | Ref | -- | 0.68 [0.48 - 0.96] | -- |
| Secondary | 0.78 [0.36 - 1.67] | 0.90 [0.60 - 1.34] | 0.89 [0.64 - 1.24] | 0.92 [0.11 - 7.40] | -- | 0.51 [0.34 - 0.76] | -- |
| Tertiary | 0.63 [0.26 - 1.56] | 0.50 [0.10 - 2.39] | 0.49 [0.25 - 0.93] | 3.33 [1.01 - 10.98] | -- | 0.36 [0.19 - 0.68] | -- |
| **(>1961)** |  |  |  |  |  |  |  |
| No schooling | -- | Ref | Ref | -- | -- | Ref | -- |
| Primary | Ref | 0.81 [0.47 - 1.39] | 1.00 [0.61 - 1.64] | -- | -- | 0.40 [0.15 - 1.09] | -- |
| Secondary | 0.49 [0.20 - 1.24] | 0.79 [0.47 - 1.31] | 0.90 [0.55 - 1.46] | -- | -- | 0.30 [0.11 - 0.83] | -- |
| Tertiary | 0.06 [0.01 - 0.47] | 0.22 [0.05 - 1.01] | 0.55 [0.23 - 1.32] | -- | -- | 0.29 [0.09 - 0.88] | -- |
| **Wealth** |  |  |  |  |  |  |  |
| **(<1940)** |  |  |  |  |  |  |  |
| Low (ref) |  |  |  |  |  |  |  |
| Medium | 0.80 [0.63 - 1.00] | 0.95 [0.80 - 1.12] | 0.82 [0.69 - 0.97] | 1.31 [0.39 - 4.39] | -- | 1.10 [0.93 - 1.30] | 1.06 [0.61 - 1.85] |
| High | 0.72 [0.57 - 0.92] | 0.79 [0.65 - 0.96] | 0.76 [0.64 - 0.90] | 0.80 [0.21 - 3.02] | -- | 0.63 [0.52 - 0.75] | 1.03 [0.58 - 1.84] |
| **(1941-1950)** |  |  |  |  |  |  |  |
| Low (ref) |  |  |  |  |  |  |  |
| Medium | 0.44 [0.21 - 0.90] | 0.72 [0.58 - 0.90] | 0.74 [0.57 - 0.95] | 2.60 [1.19 - 5.69] | -- | 0.86 [0.71 - 1.04] | 0.78 [0.35 - 1.77] |
| High | 0.41 [0.20 - 0.84] | 0.65 [0.51 - 0.82] | 0.69 [0.53 - 0.89] | 1.49 [0.60 - 3.72] | -- | 0.41 [0.33 - 0.51] | 0.21 [0.06 - 0.74] |
| **(1951-1960)** |  |  |  |  |  |  |  |
| Low (ref) |  |  |  |  |  |  |  |
| Medium | 0.62 [0.32 - 1.23] | 0.66 [0.39 - 1.11] | 0.64 [0.48 - 0.87] | 0.98 [0.25 - 3.88] | -- | 0.74 [0.55 - 0.99] | 2.69 [0.85 - 8.50] |
| High | 0.27 [0.10 - 0.68] | 0.49 [0.37 - 0.66] | 0.72 [0.54 - 0.96] | 0.44 [0.10 - 2.01] | -- | 0.24 [0.17 - 0.34] | 0.24 [0.03 - 2.17] |
| **(>1961)** |  |  |  |  |  |  |  |
| Low (ref) |  |  |  |  |  |  |  |
| Medium | 0.68 [0.26 - 1.73] | 0.67 [0.42 - 1.06] | 0.52 [0.32 - 0.84] | -- | -- | 0.70 [0.48 - 1.03] | -- |
| High | 0.90 [0.29 - 2.78] | 0.64 [0.40 - 1.02] | 0.58 [0.36 - 0.93] | -- | -- | 0.29 [0.19 - 0.46] | -- |
| **Occupation** |  |  |  |  |  |  |  |
| **(<1940)** |  |  |  |  |  |  |  |
| Manual (ref) |  |  |  |  |  |  |  |
| Farmers | -- |  | 1.25 [0.91 - 1.72] |  |  | 0.99 [0.75 - 1.30] |  |
| Lower non-manual | 0.99 [0.78 - 1.25] |  | 1.61 [1.17 - 2.21] |  |  | 1.05 [0.78 - 1.42] |  |
| Upper non-manual | 0.82 [0.65 - 1.03] |  | 1.04 [0.57 - 1.91] |  |  | 0.91 [0.62 - 1.32] |  |
| **(1941-1950)** |  |  |  |  |  |  |  |
| Manual (ref) |  |  |  |  |  |  |  |
| Farmers | -- |  | 0.90 [0.63 - 1.29] |  |  | 0.99 [0.76 - 1.29] |  |
| Lower non-manual | 0.47 [0.23 - 0.94] |  | 0.73 [0.51 - 1.05] |  |  | 0.96 [0.73 - 1.25] |  |
| Upper non-manual | 0.44 [0.22 - 0.90] |  | 1.13 [0.69 - 1.87] |  |  | 0.95 [0.68 - 1.33] |  |
| **(1951-1960)** |  |  |  |  |  |  |  |
| Manual (ref) |  |  |  |  |  |  |  |
| Farmers | -- |  | 0.77 [0.55 - 1.08] |  |  | 0.57 [0.36 - 0.91] |  |
| Lower non-manual | 1.56 [0.64 - 3.79] |  | 0.70 [0.50 - 0.98] |  |  | 0.65 [0.46 - 0.93] |  |
| Upper non-manual | 1.39 [0.63 - 3.06] |  | 0.38 [0.21 - 0.69] |  |  | 0.32 [0.19 - 0.57] |  |
| **(>1961)** |  |  |  |  |  |  |  |
| Manual (ref) |  |  |  |  |  |  |  |
| Farmers | -- |  | 1.31 [0.68 - 2.51] |  |  | 0.97 [0.42 - 2.22] |  |
| Lower non-manual | 0.24 [0.07 - 0.82] |  | 1.13 [0.63 - 2.06] |  |  | 0.78 [0.53 - 1.16] |  |
| Upper non-manual | 0.22 [0.09 - 0.57] |  | 0.47 [0.17 - 1.29] |  |  | 0.79 [0.46 - 1.36] |  |

Notes: Results are presented Hazard Ratios relative to individuals in the reference groups;

Estimates are from Cox Proportion Hazards models with relaxed assumption of the proportionality by allowing the baseline hazard functions to differ by gender.
